# Statistical driver genes as a means to uncover missing heritability for age-related macular degeneration

**DOI:** 10.1186/s12920-020-00747-4

**Published:** 2020-07-06

**Authors:** Andrea R. Waksmunski, Michelle Grunin, Tyler G. Kinzy, Robert P. Igo, Jonathan L. Haines, Jessica N. Cooke Bailey

**Affiliations:** 1 Department of Genetics and Genome Sciences, Case Western Reserve University, Cleveland, OH 44106 USA; 2 Cleveland Institute for Computational Biology, Case Western Reserve University, Cleveland, OH 44106 USA; 3 Department of Population and Quantitative Health Sciences, Case Western Reserve University, Cleveland, OH 44106 USA

**Keywords:** Genome-wide association study, Pathway analysis, Statistical driver gene, GREML, Heritability

## Abstract

**Background:**

Age-related macular degeneration (AMD) is a progressive retinal disease contributing to blindness worldwide. Multiple estimates for AMD heritability (*h*^*2*^) exist; however, a substantial proportion of *h*^*2*^ is not attributable to known genomic loci. The International AMD Genomics Consortium (IAMDGC) gathered the largest dataset of advanced AMD (ADV) cases and controls available and identified 34 loci containing 52 independent risk variants defining known AMD *h*^*2*^. To better define AMD heterogeneity, we used Pathway Analysis by Randomization Incorporating Structure (PARIS) on the IAMDGC data and identified 8 statistical driver genes (SDGs), including 2 novel SDGs not discovered by the IAMDGC. We chose to further investigate these pathway-based risk genes and determine their contribution to ADV *h*^*2*^, as well as the differential ADV subtype *h*^*2*^.

**Methods:**

We performed genomic-relatedness-based restricted maximum-likelihood (GREML) analyses on ADV, geographic atrophy (GA), and choroidal neovascularization (CNV) subtypes to investigate the *h*^*2*^ of genotyped variants on the full DNA array chip, 34 risk loci (*n* = 2758 common variants), 52 variants from the IAMDGC 2016 GWAS, and the 8 SDGs, specifically the novel 2 SDGs, *PPARA* and *PLCG2*.

**Results:**

Via GREML, full chip *h*^*2*^ was 44.05% for ADV, 46.37% for GA, and 62.03% for CNV. The lead 52 variants’ *h*^*2*^ (ADV: 14.52%, GA: 8.02%, CNV: 13.62%) and 34 loci *h*^*2*^ (ADV: 13.73%, GA: 8.81%, CNV: 12.89%) indicate that known variants contribute ~ 14% to ADV *h*^*2*^. SDG variants account for a small percentage of ADV, GA, and CNV heritability, but estimates based on the combination of SDGs and the 34 known loci are similar to those calculated for known loci alone. We identified modest epistatic interactions among variants in the 2 SDGs and the 52 IAMDGC variants, including modest interactions between variants in *PPARA* and *PLCG2*.

**Conclusions:**

Pathway analyses, which leverage biological relationships among genes in a pathway, may be useful in identifying additional loci that contribute to the heritability of complex disorders in a non-additive manner. Heritability analyses of these loci, especially amongst disease subtypes, may provide clues to the importance of specific genes to the genetic architecture of AMD.

## Background

Genome-wide association studies (GWAS) have been instrumental in identifying genomic variants associated with complex traits for over 10 years [1]. GWAS detect such associations by comparing allele frequencies in individuals with and without a trait of interest in a specific population [2]. These methods have been successfully applied to find large numbers of disease-associated variants that contribute to the trait’s heritability [3]. Heritability is defined as the fraction of phenotypic variance explained by genetic variation in the context of a specific range of environmental variation [3]. Broad-sense heritability (*H*^*2*^) is the proportion of phenotypic variation that includes dominance and epistasis; whereas, narrow-sense heritability (*h*^*2*^) is the proportion of phenotypic variation of additive genetic effects. Common variants may capture up to about two-thirds of narrow-sense heritability for age-related macular degeneration (AMD), but despite this, much of AMD heritability is still unexplained by known variants [4]. The topic of missing heritability has been discussed, especially regarding complex diseases, and may be attributable to non-additive effects of genomic variants that are not discernible from traditional GWAS [3].

GWAS have been remarkably successful for identifying genomic loci contributing substantially to AMD risk. This progressive, adult-onset condition is among the leading causes of blindness in the world in individuals over 60 and is expected to become a significant health burden as the aging population increases in size [5]. AMD leads to the decline of central vision in patients as a result of lipid deposits (drusen), photoreceptor loss, and inflammation in the macula [6, 7]. It is clinically characterized into multiple subtypes: early, intermediate, or advanced AMD stages based on disease severity. Advanced AMD (ADV) is further sub-categorized into geographic atrophy (GA) or choroidal neovascularization (CNV).

The International AMD Genomics Consortium (IAMDGC) performed the largest case-control GWAS to date for ADV and identified 52 independent common and rare variants from 34 susceptibility loci, the highest risk loci being the *CFH* and *ARMS2/HTRA1* genes [8]. These 52 genomic variants contribute to about half of the genomic heritability for ADV, which leaves nearly half of ADV heritability unexplained [8].

In contrast to traditional case-control GWAS, in silico pathway analyses of GWAS summary statistics identify biological pathways, which are defined by interactions of genes for a common biological function, harboring excesses of genomic variants that may be associated with a trait [9, 10]. They accomplish this by grouping variants into features that are then merged into pathways based on curated information in publicly available pathway databases [9, 10]. Because pathway analyses focus broadly on the collection of nominal genetic variants in biological pathways, they are not limited to assessing additive effects of individual variants on the trait and may be leveraged to identify genetic variance with non-additive effects. Ultimately, these analyses provide insights into trait-associated biological processes and suggest which genes are most pertinent for these pathway-level associations [11, 12]. However, they do not estimate the contribution of genetic variants in these genes and pathways to the trait’s heritability [13].

To uncover genomic loci undetectable by GWAS, we performed in silico, knowledge-driven pathway analyses of the summary statistics from the IAMDGC 2016 GWAS [14] using Pathway Analysis by Randomization Incorporating Structure (PARIS) [11, 12]. In our comprehensive approach, we utilized multiple pathway databases to determine which genes were consistently contributing to significant pathway signals for ADV [14]. We identified eight statistical driver genes (SDGs) that were significantly contributing to the significant AMD-associated pathways from PARIS: *C2, C3, LIPC, MICA, NOTCH4, PLCG2, PPARA,* and *RAD51B.* Of these eight SDGs, two genes (*PLCG2* and *PPARA*) fell outside of the 34 AMD susceptibility loci identified by the IAMDGC GWAS [8]; we showed that these loci may be associated with ADV risk [14].

While the 2016 IAMDGC GWAS uncovered several AMD loci that explain a large portion of AMD heritability [8], their study did not investigate potential non-additive effects of AMD risk genes. Pathway analyses of GWAS data consider known biological relationships among genes in a pathway; therefore, we were able to identify two novel AMD genes (*PLCG2* and *PPARA*) that were not found in the IAMDGC GWAS. To further examine the potential role of the 2 novel SDGs, we calculated the proportion of ADV variance explained by (i) common variants in *PPARA* and *PLCG2*, (ii) the 8 SDGs identified by pathway analysis, and (iii) known and novel AMD loci identified by the IAMDGC. We also applied this approach to the subtypes of ADV (GA and CNV) to elucidate whether these variants contribute more to the heritability of AMD in a particular subtype of ADV. We further interrogated the possible epistatic interactions among lead variants in the known AMD genes as well as our novel SDGs to elucidate if their contributions to AMD heritability could be attributable to non-additive effects.

## Methods

### Statistical driver genes for advanced AMD

We performed in silico pathway analyses using Pathway Analysis by Randomization Incorporating Structure (PARIS, v2.4) [12] on the largest available ADV case-control GWAS results from the IAMDGC [8]. This included summary statistics for 445,115 directly genotyped variants on 16,144 advanced AMD cases and 17,832 controls [8]. The ADV cases include GA-specific cases (*n* = 3235), CNV-specific cases (*n* = 10,749), and individuals with both GA and CNV (*n* = 2160) [8]. Samples were genotyped with the Illumina HumanCoreExome Array as previously described [8] and are accessible through the database of Genotypes and Phenotypes (dbGAP; Accession: phs001039.v1.p1). Our knowledge-driven pathway analyses utilized three pathway databases (KEGG, Reactome, and GO) and led to the discovery of eight statistical driver genes for ADV (Table 1) [14]*.* Statistical driver genes (SDGs) were defined as genes that were strongly contributing (gene-level *p* < 0.0001) to the statistical signal of the significant pathways (pathway-level *p* < 0.0001) identified by PARIS. Two of these SDGs (*PPARA* and *PLCG2*) remained significant following the exclusion of the 34 known AMD loci identified by the IAMDGC from the pathway analysis because they fall outside of the known loci boundaries.

**Table 1:** Statistical driver genes for advanced AMD identified with PARIS

Gene	Chromosome	Full Gene Name
*C2*	6	complement C2
*MICA*	6	MHC class I polypeptide-related sequence A
*NOTCH4*	6	notch receptor 4
*RAD51B*	14	RAD51 paralog B
*LIPC*	15	lipase C, hepatic type
***PLCG2***	**16**	**phospholipase C gamma 2**
*C3*	19	complement C3
***PPARA***	**22**	**peroxisome proliferator activated receptor alpha**

The eight statistical driver genes were identified with pathway analysis using PARIS and multiple biological pathway databases [14]. Two of these genes (*PLCG2* and *PPARA*) were not previously identified as a part of the IAMDGC susceptibility loci and are noted in bold. Full gene names are based on the HUGO Gene Nomenclature Committee (HGNC)

### Variant selection and genotype extraction

For our heritability estimates, we extracted genotypes for variants in one of these seven variant criteria subsets (Table 3):

- IAMDGC Chip: Variants that were directly genotyped by the IAMDGC on the Illumina HumanCoreExome chip with custom content as previously described [8]
- 8 SDGs ±50 kb: Variants in or within 50 kilobasepairs (kb) of the eight SDGs (*C2, C3, LIPC, MICA, NOTCH4, PLCG2, PPARA,* and *RAD51B*) [14]
- 2 Novel SDGs ±50 kb: Variants in or within 50 kb of the *PLCG2* and *PPARA* genes [14]
- Lead IAMDGC Variants: Variants that were identified as one of the 52 lead variants from the IAMDGC 2016 GWAS [8]
- 34 AMD Loci: Variants in the 34 susceptibility loci identified by the IAMDGC 2016 GWAS (Supplementary Table 5 in [8])
- 34 AMD Loci and 2 Novel SDGs ±50 kb: Variants that occur in the 34 susceptibility loci identified by the IAMDGC 2016 GWAS (Supplementary Table 5 in [8]) and variants that fall in or within 50 kb of the *PLCG2* and *PPARA* genes [14]
- 34 AMD Loci and 8 SDGs ±50 kb: Variants in the 34 susceptibility loci identified by the IAMDGC 2016 GWAS (Supplementary Table 5 in [8]) and variants that fall in or within 50 kb of the eight SDGs [14]

Gene and loci boundaries were based on build GRCh37 of the human genome. Using PLINK v1.90 beta [15, 16], we filtered the variants from the 34 loci and SDGs based on minor allele frequency (MAF) and genotyping call rate to exclude variants that had MAF < 0.01 and missing genotype rate > 0.01. The variants from the IAMDGC chip and the 52 lead variants were not filtered for MAF or call rate; therefore, these two variant sets include common and rare variants. Penetrance was not taken into account for the SDGs [14] because their association was based on summary statistics from the IAMDGC 2016 GWAS. All variant sets were extracted from the ADV case-control data, GA-specific case-control data (GA), and CNV-specific case-control data (CNV) (Table 2).

**Table 2:** Demographics of participants in the study data

Data	ADV	GA	CNV
Total Samples	33,976	21,067	28,581
Cases	16,144	3235	10,749
Controls	17,832	17,832	17,832

Values represent counts of samples in the IAMDGC data

### Estimation of AMD heritability with GCTA GREML

Genetic relationship matrices (GRMs) were constructed using Genome-wide Complex Trait Analysis (GCTA v1.91.3beta) [17] for each category of late AMD disease states (ADV (GA and CNV combined), GA, and CNV), and for each subset of variants we selected (Tables 2 and 3, respectively). This included variants within *PPARA* and *PLCG2*, variants within 8 previously identified SDGs, the 34 loci, the 52 lead IAMDGC variants, and the full directly genotyped IAMDGC chip. To obtain invertible and reliable GRMs, variants were filtered by minor allele frequency (MAF < 0.01) and missingness (missing genotype rate > 0.01) when constructing the GRMs for the 34 AMD loci and the SDGs. Without this filtering step, GCTA is unable to create functional (invertible) GRMs for subsequent restricted maximum-likelihood (REML) analyses, which estimate the proportion of phenotypic variance attributable to additive genetic variance [17].

**Table 3:** Characteristics of the marker data extracted from the IAMDGC exome chip

Data	ADV	GA	CNV
IAMDGC Chip	553,261	553,261	553,261
8 SDGs ±50 kb	1122	1122	1122
2 Novel SDGs ±50 kb	79	79	79
Lead IAMDGC Variants	52	52	52
34 AMD Loci	2758	2758	2758
34 AMD Loci and 2 Novel SDGs ±50 kb	2835	2992	2992
34 AMD Loci and 8 SDGs ±50 kb	3351	4411	3351

Values represent counts of pruned variants based on MAF and call rate. The 8 SDGs are *C2, C3, LIPC, MICA, NOTCH4, PLCG2, PPARA,* and *RAD51B*. The 2 novel SDGs are *PLCG2* and *PPARA*. The different numbers of variants for the expanded loci analyses were attributable to the different MAFs observed for each of the advanced AMD subtypes: ADV, GA, and CNV

We used the genomic-relatedness-based restricted maximum-likelihood (GREML) approach in the GCTA v1.91.3beta software to estimate narrow-sense heritability (*h*^*2*^) for each of the three advanced AMD datasets (ADV, GA, and CNV) and for each of the 7 variant sets. The GREML approach does not measure dominance variance or epistatic interactions. We also estimated chip heritability adjusting for age information available from the IAMDGC (age at diagnosis for cases and age at exam for controls) and ten principal components (PC) in the GREML analysis of the full chip. The ten PCs were calculated from genome-wide chip data using PLINK v1.90 beta [15, 16], which is a port of GCTA (https://www.cog-genomics.org/plink/1.9/strat). We also estimated *h*^*2*^ based on a population prevalence of ADV in individuals of European descent (0.5%, [5]), as the IAMDGC samples in this study were filtered to unrelated individuals of European ancestry [8]. We validated that the estimates we observed were not likely artifacts by re-performing our analyses of the ADV data and a random set of 1112 variants (equal to the number of common variants in the 8 SDGs) that met the same filtering criteria for common variants in the 8 SDGs. In addition, we performed the same analysis using random sets of 79 variants (equal to the number of variants in the 2 SDGs alone) that met the same filtering criteria for the common variants in the 2 SDGs to validate that the estimates we observed were not likely to be artifacts.

### Pairwise LD analysis of SDGs and 34 AMD loci

To evaluate the linkage and independence of the variants in the 8 SDGs and the 34 AMD loci (Table 3), we examined pairwise linkage disequilibrium (LD) using two computational tools: LDMatrix [18] and SNiPA [19]. Variant pairs with *r*^2^ > 0.7 were determined to be the same signal and not considered in downstream assessments. We further assessed whether or not each variant in the pair was considered part of an SDG, one of the AMD loci, or overlapped between the two groups of variants.

### Epistatic interaction analyses

To investigate heritability attributable to non-additive interactions among the lead AMD variants from the IAMDGC GWAS [8] and the novel SDGs [14], we performed pairwise logistic regression-based epistasis analyses using PLINK v1.90 beta [15, 16]. Analyses were performed over the full set of variants in or within 50 kb of the 2 SDGs and the 52 lead variants from the 2016 IAMDGC GWAS [8]; therefore, the threshold for significance was set at 2.31 × 10^− 6^ for multiple testing correction (Bonferroni correction for 21,675 tests). If both variants in an epistatic interaction were from the same gene/locus, we found their LD in the European population using LDlink (https://ldlink.nci.nih.gov/, [18]). If the r^2^ was greater than 0.7, we determined the signals to be the same.

## Results

### Study data for ADV, GA, and CNV analyses

We aimed to determine the proportion of ADV, GA-specific, and CNV-specific heritability explained by variants in and within 50 kilobasepairs (kb) of the SDGs identified by PARIS (Tables 2 and 3). We extracted 2173 variants from all 8 previously identified SDGs and 234 variants from the 2 novel SDGs (*PPARA* and *PLCG2*) based on their gene boundaries (Methods). However, we found that several of the variants in the SDGs either had very low MAF or had a low genotype call rate in the samples we analyzed and, therefore, they were removed prior to GRM creation (Table 3).

### Narrow-sense heritability explained by variants in SDGs for ADV, GA, and CNV

To determine whether the SDGs contribute to the missing heritability of ADV or its subtypes (GA and CNV), we performed GREML analyses of variants from the SDGs. We found that the percent of ADV risk explained by the common, high call rate variants in the 8 SDGs was 3.76% (S.E. = 0.39) (Table 4). This was higher than the estimate observed for GA and comparable to CNV *h*^*2*^ estimate (2.53 and 3.71%, respectively) (Table 4). The 2 SDGs contribute to 0.097, 0.12, and 0.18% ADV, GA, and CNV risk, respectively (Table 4).

**Table 4:** Heritability estimates for advanced AMD (ADV), GA, and CNV based on our variant sets

Data	ADV		GA		CNV	
	Estimate (%)	S.E. (%)	Estimate (%)	S.E. (%)	Estimate (%)	S.E. (%)
**IAMDGC Chip**	44.05	1.29	46.37	2.13	62.03	1.47
**8 SDGs ± 50 kb**	3.76	0.39	2.53	0.36	3.71	0.41
**2 Novel SDGs ± 50 kb**	0.097	0.048	0.12	0.076	0.18	0.076
**Lead IAMDGC Variants**	14.52	2.48	8.02	1.50	13.62	2.36
**34 AMD Loci**	13.73	0.83	8.81	0.75	12.89	0.82
**34 AMD Loci and 2 Novel SDGs ± 50 kb**	13.51	0.81	8.60	0.70	12.20	0.74
**34 AMD Loci and 8 SDGs ± 50 kb**	13.06	0.73	8.78	0.63	12.59	0.74

For the analyses including the SDGs and/or 34 loci, we pruned the variants based on minor allele frequency and genotype call rate in the samples. For the estimates calculated for the full IAMDGC chip and lead IAMDGC variants, we did not apply variant filtering. kb: kilobasepairs

To compare the SDG heritability estimates for ADV, GA, and CNV to those observed for known AMD loci, we performed GREML analyses of the common variants with high call rates within the 34 loci identified by the IAMDGC [8] for each disease subtype. We found that common variants from the known loci contribute to 13.73% (S.E. = 0.83) ADV risk, 8.81% (S.E. = 0.75) GA risk, and 12.89% (SE = 0.82) CNV risk (Table 4). The 52 lead variants identified by the IAMDGC alone explain 14.52% (S.E. = 2.48) of ADV risk. By comparison, the *h*^*2*^ estimates for these variants were lower (8.02 and 13.62%) for GA and CNV, respectively.

Given the individual estimates for the SDGs and 34 loci, we performed GREML analyses on combinations of the SDGs and 34 loci for ADV, GA, and CNV. In all our analyses, we found that the *h*^*2*^ estimates were very similar (Table 4). Together, the 34 loci and 8 SDGs contribute to 13.06, 8.78, and 12.59% *h*^*2*^ for ADV, GA, and CNV, respectively (Table 4). The *h*^*2*^ estimates derived from the 34 loci and 2 SDGs are comparable to these values (ADV: 13.51%, GA: 8.60%, and CNV: 12.20%) (Table 4). To interrogate existing linkage among the SDG variants and those in the 34 AMD loci, we performed pairwise linkage disequilibrium (LD) analyses of these variants. No pairs of variants in high LD (*r*^2^ > 0.7) were found between any of the variants in the 8 SDGs and the expanded 34 loci. This indicates that the variants we compared did not have pre-existing LD outside of the known AMD loci and were independent of each other.

To replicate chip heritability calculations from the 2016 GWAS published by the IAMDGC, we performed GREML analyses of the full datasets (i.e. chip heritability) for ADV, GA, and CNV. The chip heritability for ADV was 44.05% (S.E. = 1.29) (Table 5). We achieved similar values (44.16% (S.E. = 1.29)) when we re-performed our analyses with the first 10 principal components (PCs) calculated for the full chip and ADV cases and controls. Chip heritability estimates were higher for GA and CNV (46.37 and 62.03%, respectively) than the heritability estimate for the combined ADV dataset (Table 5). These values were similar to the estimates calculated for GA and CNV chip heritability including PCs for those respective datasets (46.50 and 62.18%, respectively). Values decreased after incorporating age data available from the IAMDGC (age at diagnosis for cases and age at exam for controls) and the first 10 PCs into the GREML analyses for chip heritability of the ADV and its subtypes (Table 5).

**Table 5:** Chip heritability estimates calculated by GREML for advanced AMD (ADV), GA, and CNV using quantitative covariates

Analyses	ADV		GA		CNV	
	Estimate (%)	S.E. (%)	Estimate (%)	S.E. (%)	Estimate (%)	S.E. (%)
**Full Chip (no covariates)**	44.05	1.29	46.37	2.13	62.03	1.47
**With Age**	57.96	1.31	45.41	2.16	59.71	1.51
**With 10 PCs**	44.16	1.29	46.50	2.13	62.18	1.48
**With Age + 10 PCs**	42.06	1.31	41.24	1.97	60.21	1.51

These estimates were based on 15,656 advanced AMD (ADV) cases (2964 GA-specific cases and 10,340 CNV-specific cases) and 17,832 controls with age information in the IAMDGC data or with an age over 50. Covariates included age information available from the IAMDGC (age at diagnosis for cases and age at exam for controls) and 10 PCs calculated for each of the datasets

To further verify that contributions to ADV heritability from the common variants in the 8 SDGs were unlikely due to chance, we selected randomized variants from the autosomal genome that met the same MAF criteria we had used before. We recreated the GRM for the ADV data and these variants and performed the same GREML analyses we had on the 8 SDGs. The 1122 randomized variants explain 1.76% (S.E. = 0.21), indicating that the ADV heritability estimate for the 8 SDG (3.76%) was not likely due to chance. In addition, we ran several (> 5) randomized variant analyses with different sets of 79 random variants to mimic the number of variants in the 2 novel SDGs and found that they explain at most 0.076% (S.E. = 0.048). Although this is near the estimate we calculated for the 2 SDGs alone (0.097%), it is unlikely that the ADV heritability estimate for the 2 SDGs alone (0.097%) was due to chance.

### Epistasis analyses

To interrogate possible interactions among the 52 AMD variants identified by the IAMDGC and the variants in the novel SDGs (*PPARA* and *PLCG2*), we performed logistic regression-based epistasis analyses using PLINK [15, 16]. Although we did not identify any significant interactions, several modest epistatic interactions (*p* < 0.001) were uncovered between known AMD variants and variants in *PPARA* and *PLCG2* (Table 6). Interactions between *PPARA* and *PLCG2* variants were also identified but did not reach the significance threshold correcting for multiple testing (Table 7).

**Table 6:** Epistatic interactions from pairwise logistic regression-based epistasis testing between variants in or within 50 kb of the 2 novel SDGs (*PPARA* and *PLCG2*) and the 52 AMD-associated index variants from the 2016 IAMDGC GWAS

AMD Locus from IAMDGC GWAS				AMD SDG Locus				Epistatic Interaction	
Chr	Location (bp)	rsID	Locus Name	Chr	Location (bp)	rsID	Locus Name	OR	***P***
1	196,704,632	rs10922109	***CFH***	22	46,681,141	rs35883013	***PPARA***	1.55	0.00015
9	76,617,720	rs10781182	***MIR6130/RORB***	16	81,932,165	rs4889432	***PLCG2***	1.09	0.00048
5	39,327,888	rs62358361	***C9***	16	81,821,531	rs8043845	***PLCG2***	0.66	0.00073
14	68,769,199	rs61985136	***RAD51B***	16	81,900,628	rs4243218	***PLCG2***	1.08	0.00079
6	3,255,581	rs204993	***C2/C3/CFB***	22	46,685,754	rs182313981	***PPARA***	0.63	0.00093
19	45,411,941	rs429358	*APOE*	22	46,685,754	rs182313981	*PPARA*	0.53	0.00099

Locations are given in base pairs (bp) based on build 37 of the human genome. None of these interactions are significant after correcting for multiple testing (*p* < 2.31 × 10^− 6^). Chr: Chromosome, *P: p*-values from the logistic regression test, distributed as χ^2^ with 1 d.f

**Table 7:** Epistatic interactions between variants in or within 50 kb of *PLCG2* and *PPARA* from pairwise logistic regression-based epistasis testing

***PLG2*** Variants				***PPARA*** Variants				Epistatic Interaction	
Chr	Location (bp)	rsID	Locus Name	Chr	Location (bp)	rsID	Locus Name	OR	***P***
16	81,979,125	rs12921780	***PLCG2***	22	46,633,037	rs41479847	***PPARA***	1.38	0.00075
16	81,817,239	rs4580153	***PLCG2***	22	46,638,486	rs78864133	***PPARA***	1.16	0.00095

Locations are given in base pairs (bp) based on build 37 of the human genome. Neither of these interactions are significant after correcting for multiple testing (*p* < 2.31 × 10^−6^). Chr: Chromosome, *P: p*-values from the logistic regression test, distributed as χ^2^ with 1 d.f

## Discussion

In this study, we estimated the proportion of ADV heritability attributable to the SDGs we previously identified by pathway analysis of the summary statistics from the IAMDGC 2016 GWAS [14]. This included common variants from the 8 SDGs that exhibited significant signals across significant pathways (*p* < 0.0001) from KEGG, Reactome, and GO pathway databases and the 2 SDGs (*PPARA* and *PLCG2*) that fell outside of the known AMD loci identified by the IAMDGC in their recent GWAS. To compare our results with those obtained by the IAMDGC [8], we calculated heritability estimates for the whole DNA array chip, 34 AMD loci, 52 lead variants from the 34 loci, and combinations of the SDGs and the 34 loci. The estimates and 95% confidence intervals we calculated for ADV (41.52–46.58%) and GA (42.20–50.54%) chip heritability overlap with the 95% confidence intervals for chip heritability determined by the IAMDGC for ADV (44.5–48.8%, [8]) and GA (47.2–57.4%, [8]). By contrast, our estimate for CNV chip heritability (59.15–64.91%) was much higher than what was calculated by the IAMDGC (42.2–46.5%), [8]).

Our estimate for ADV heritability based on the 52 lead variants is lower than that observed for these variants by the IAMDGC (14.52% vs. 27.2%, respectively) [8]. This difference is likely due to the different methods used to estimate these values. The IAMDGC [8] calculated their estimates by a theoretical, population-based formula based on the log odds ratios, allele frequencies of the 52 variants and the assumed trait prevalence. This formula assumes that all markers are independent, and therefore that all contributions to genetic variance are additive [20]. The IAMDGC also assumed disease prevalence of 1, 5%, or 10% in their analyses [8]. The addition of the two lead variants of the *PLCG2* and *PPARA* genes does not increase our heritability estimates for ADV (14.54%). These results reinforce the notion that the variants in these SDGs in isolate are not significant but in aggregate contribute strongly to the statistical signals we previously observed for AMD-associated pathways. Their association with AMD is likely not additive but rather as a consequence of their interactions within AMD-associated pathways, demonstrating the benefit of using pathway analysis to identify genetic variance with non-additive effects.

Heritability estimates based on each of our variant sets varied based on the advanced AMD subtype analyzed (ADV, GA, or CNV). With the exception of the chip heritability estimates, the values estimated for ADV and CNV were much higher than those calculated for GA. This may be due to the lower sample size of GA cases in the dataset. Based on the GCTA GREML power calculator (http://cnsgenomics.com/shiny/gctaPower/), we had good (over 80%) power to detect genetic variance for GA. It has been previously shown that particular AMD-associated variants contribute to a particular subtype. For instance, the IAMDGC identified the first subtype-specific variant for CNV near the *MMP9* gene on chromosome 20 [8]. Additional genes involved in extracellular matrix maintenance have been implicated in ADV subtypes, not intermediate AMD [21]. Although the *HTRA1/ARMS2* locus contributes generally to ADV risk (including both subtypes), it has been consistently associated with increased CNV-specific risk [22–25], and smokers with the Y402H risk allele of *CFH* have an increased risk of developing wet AMD specifically [26].

Based on our calculations of the heritability explained collectively by the SDGs and the 34 AMD loci, we hypothesize that the contributions of the common variants in these regions may not be purely additive. Additionally, we suspect that that the contributions of the common variants in *PPARA* and *PLCG2* drive the heritability estimates for the combinations of variants we tested given the nearly identical estimates for the combination of the common variants from the 8 SDGs and 34 loci relative to the combination of the common variants from the 2 SDGs and 34 loci. In the IAMDGC GWAS, the locus boundaries were defined by distance and LD structure from the lead variant in each locus [8]. Therefore, based on the definitions of the 8 SDGs and 34 loci, we expanded the amount of variants covered in 6 of the 34 loci (Table 3) in our combined analysis (34 AMD Loci and 8 SDGs ±50 kb) in this study. Based on our pairwise disequilibrium analysis, these additional variants are mostly independent of the variants in the known loci. Only a few variants were in LD (*r*^2^ > 0.7), but these variants were only connected to one variant in an SDG locus. By contrast, in the analysis of the common variants from the 34 loci and 2 SDGs, the 34 loci themselves were not expanded despite the addition of the 2 SDGs.

The variance explained by genetic variants in genes from AMD-related pathway defined in the literature has been previously explored using GCTA [13]. The 19 then-known AMD associated variants explained 13.3% of AMD risk in general, and significant additional heritability was attributable to variants in inflammatory and complement pathways when accounting for the known risk variants [13]. Other pathways, including angiogenesis and apoptosis, did not significantly contribute to AMD heritability estimates [13]. By contrast, in our approach, pathways were identified via in silico pathway analysis of large-scale GWAS data with PARIS and multiple curated pathway databases. We then focused specifically on the SDGs that significantly contribute to AMD-associated pathways in our analysis. Gene expression profiles for the two novel SDGs (*PLCG2* and *PPARA*) have been observed in retinas from AMD cases and controls [27]. In retinal tissue from unaffected individuals, *PPARA* is highly expressed; whereas, *PLCG2* is weakly expressed [28, 29]. Neither of these genes were significantly expressed in an age-adjusted analysis of CNV retinas [27].

While we determined that common variants from the SDGs contribute to the ADV, GA, and CNV heritability, this study had several limitations. Even with our estimates, there is a substantial portion of ADV, GA, and CNV variance left unexplained by the loci interrogated in this study. Additional sources of heritability not examined in this study include rare variants, structural variants, further investigations into epistasis, and epigenetic effects. Seven rare variants were among the 52 independent, genome-wide significant markers identified by the IAMDGC in their recent GWAS [8]. In this study, we excluded rare variants (MAF < 1%) from our GREML analyses for the known loci and the SDGs, removing about half of variants from the 8 SDG variants and about two-thirds of the variants from *PPARA* and *PLCG2* alone. Rare variants were not excluded in our previous pathway analyses because PARIS does not take MAF into account when identifying associated pathways [11, 12]. Therefore, we were unable to consider the complete contributions of these variants to disease heritability. Additionally, the IAMDGC data repository we utilized in this study does not currently have further information on environmental exposures or behaviors of the study participants that may contribute to AMD risk, such as smoking status and diet. Therefore, these non-genetic factors could not be included in our study.

Given the non-additive nature of our heritability estimates for the combinations of known AMD loci and the SDGs for ADV and its subtypes, we hypothesize that the variants in these loci may be interacting with one another. Our epistasis analyses did not reveal any significant epistatic interactions among the known AMD variants and the variants in the 2 novel SDGs. However, this is only an initial look at the possible representation of epistatic interactions between these genes. We currently only examined epistatic effects directly between the common, high call rate variants in the 2 novel SDG and the 52 lead variants from the IAMDGC; therefore there could be additional epistatic interactions among other variants in *PPARA* and *PLCG2* with the 34 AMD loci. In addition, large-scale genome-wide epistatic effects have not been explored. Our identification of modest interactions among these loci, including between the novel SDGs, suggests that there may be region-wide interactions that are individually too weak to discover using these analyses. Further studies should be performed to confirm potential epistatic interactions between variants in the known loci and the SDGs. Additionally, because we performed our analyses on the largest dataset of ADV cases and controls currently available, we are unable to replicate our findings with a comparable, independent dataset. As with many genetic studies, our study only included individuals of European descent. Additional work should be done to elucidate the contributions of the SDGs to AMD heritability in diverse populations because different populations may have different heritability estimates for ADV and its subtypes [30, 31].

## Conclusions

Our study elucidated the contribution of pathway SDGs and known AMD loci to the heritability of ADV and its subtypes. Heritability estimates for particular ADV subtypes were previously uncharacterized. The SDGs we analyzed in this study were previously identified from pathway analyses utilizing multiple pathway databases. This more comprehensive approach uncovered an appreciable portion of ADV heritability that had not been previously characterized. While they do not demonstrate an additive amount of heritability to that estimated for the 34 AMD susceptibility loci identified by the IAMDGC, we suspect that this is due to interaction effects or the exclusion of rare variants from our analyses. It has been previously shown that additional AMD loci (*RLBP1* and *CLUL1*) can be identified by accounting for gene x age interaction effects [32]. We propose that identifying statistical driver genes from in silico pathway analyses of GWAS data may be a valid approach to recognizing patterns of heritability (including non-additive contributions) from large-scale genomic data that are undetectable by GWAS. We applied this approach to ADV and its subtypes, but it could be applied to uncover novel loci associated to other complex traits for which GWAS have been performed. Additionally, pathway analysis provides biological context for the loci in GWAS, which could aid in understanding the underlying mechanisms of traits and developing targeted treatments for diseases.

## Data Availability

The genotype data analyzed during the current study were generated by the IAMDGC as previously described [8] and are available through the database of Genotypes and Phenotypes (dbGAP; Accession: phs001039.v1.p1).

## Abbreviations

dbGAP: database of Genotypes and Phenotypes
ADV: Advanced age-related macular degeneration
AMD: Age-related macular degeneration
CNV: Choroidal neovascularization
GA: Geographic atrophy
GCTA: Genome-wide Complex Trait Analysis
GREML: Genomic-relatedness-based restricted maximum-likelihood
GRM: Genetic relationship matrix
GWAS: Genome-wide association study
*h*^*2*^: Narrow-sense heritability
*H*^*2*^: Broad-sense heritability
IAMDGC: International Age-related Macular Degeneration Genomics Consortium
kb: Kilobasepairs
LD: Linkage disequilibrium
MAF: Minor allele frequency
PARIS: Pathway Analysis by Randomization Incorporating Structure
PC: Principal component
REML: Restricted maximum-likelihood
SDG: Statistical driver gene
